# Randomized trial of community health worker-led decision coaching to promote shared decision-making for prostate cancer screening among Black male patients and their providers

**DOI:** 10.1186/s13063-021-05064-4

**Published:** 2021-02-10

**Authors:** Danil V. Makarov, Zachary Feuer, Shannon Ciprut, Natalia Martinez Lopez, Angela Fagerlin, Michele Shedlin, Heather T. Gold, Huilin Li, Gina Lynch, Rueben Warren, Peter Ubel, Joseph E. Ravenell

**Affiliations:** 1 VA New York Harbor Healthcare System, 423 E 23rd St, New York, NY USA; 2 Departments of Urology, NYU Langone Health, 227 E 30th St, New York, NY USA; 3 Population Health, NYU Langone Health, 227 E 30th St, New York, NY USA; 4 Department of Population Health Sciences, University of Utah School of Medicine, Salt Lake City, UT USA; 5 NYU College of Nursing, 433 First Avenue, New York, NY USA; 6 Sunset Park Health Council, Brooklyn, NY USA; 7 National Center for Bioethics in Research and Health Care, Tuskegee University, Tuskegee, USA; 8 The Fuqua School of Business, Duke University, Durham, NC USA

**Keywords:** Prostate cancer, PSA, Racial disparity, Screening, Shared decision-making, Community health worker, Randomized controlled trial

## Abstract

**Background:**

Black men are disproportionately affected by prostate cancer, the most common non-cutaneous malignancy among men in the USA. The United States Preventive Services Task Force (USPSTF) encourages prostate-specific antigen (PSA) testing decisions to be based on shared decision-making (SDM) clinician professional judgment, and patient preferences. However, evidence suggests that SDM is underutilized in clinical practice, especially among the most vulnerable patients. The purpose of this study is to evaluate the efficacy of a community health worker (CHW)-led decision-coaching program to facilitate SDM for prostate cancer screening among Black men in the primary care setting, with the ultimate aim of improving/optimizing decision quality.

**Methods:**

We proposed a CHW-led decision-coaching program to facilitate SDM for prostate cancer screening discussions in Black men at a primary care FQHC. This study enrolled Black men who were patients at the participating clinical site and up to 15 providers who cared for them. We estimated to recruit 228 participants, ages 40–69 to be randomized to either (1) a decision aid along with decision coaching on PSA screening from a CHW or (2) receiving a decision aid along with CHW-led interaction on modifying dietary and lifestyle to serve as an attention control. The independent randomization process was implemented within each provider and we controlled for age by dividing patients into two strata: 40–54 years and 55–69 years. This sample size sufficiently powered the detection differences in the primary study outcomes: knowledge, indicative of decision quality, and differences in PSA screening rates.

Primary outcome measures for patients will be decision quality and decision regarding whether to undergo PSA screening. Primary outcome measures for providers will be acceptability and feasibility of the intervention. We will examine how decision coaching about prostate cancer screening impact patient-provider communication. These outcomes will be analyzed quantitatively through objective, validated scales and qualitatively through semi-structured, in-depth interviews, and thematic analysis of clinical encounters. Through a conceptual model combining elements of the Preventative Health Care Model (PHM) and Informed Decision-Making Model, we hypothesize that the prostate cancer screening decision coaching intervention will result in a preference-congruent decision and decisional satisfaction. We also hypothesize that this intervention will improve physician satisfaction with counseling patients about prostate cancer screening.

**Discussion:**

Decision coaching is an evidence-based approach to improve decision quality in many clinical contexts, but its efficacy is incompletely explored for PSA screening among Black men in primary care. Our proposal to evaluate a CHW-led decision-coaching program for PSA screening has high potential for scalability and public health impact. Our results will determine the efficacy, cost-effectiveness, and sustainability of a CHW intervention in a community clinic setting in order to inform subsequent widespread dissemination, a critical research area highlighted by USPSTF.

**Trial registration:**

The trial was registered prospectively with the National Institute of Health registry (www.clinicaltrials.gov), registration number NCT03726320, on October 31, 2018.

**Supplementary Information:**

The online version contains supplementary material available at 10.1186/s13063-021-05064-4.

## Background

Prostate cancer is the second leading cause of cancer death among men in the US [1] and harms Black men disproportionately [2]. Prostate cancer comprises nearly one third of all new cancers in Black men, affecting almost 30,000 men each year. The incidence rate is 70% higher for Black men compared with other racial and ethnic groups, and mortality is 2–3 times higher compared with White men. Low socioeconomic status has been found to be a predictor of poor prostate cancer outcomes, including mortality; however, the Black-White disparity persists even when accounting for all of these other factors [3].

Prostate cancer mortality has declined since the adoption of PSA screening. While some of this benefit is a result of screening, the exact effect is difficult to quantify [4, 5]. Although screening unquestionably detects many early-stage cancers, over-diagnosis of indolent cancers, leading to over-treatment, remains a significant concern [6, 7]. The USPSTF recommends an informed decision-making, or “shared decision-making” (SDM) approach, to PSA screening between men aged 55 to 69 years and their healthcare providers, and other organizations recommend screening Black men at age 40 [8]. SDM includes three critical steps—information exchange, deliberation, and shared decision—rooted in the ethical principles of autonomy (respect for a patient’s opinions and choices), justice (“what is deserved”), and beneficence (actions to maximize benefit and minimize harm) [9, 10].

While many groups advocate for SDM, including the Institute of Medicine [11] and USPSTF [12], it is rarely achieved in clinical practice [13, 14]. A nationally representative study of men considering PSA screening reported that only half were asked their preferences, and the pros and cons of screening were discussed only 32% of the time [15, 16]. These studies suggest most prostate cancer screening decisions do not meet criteria for SDM as they lack a balanced discussion of decision consequences and preference clarification. SDM is particularly important for PSA screening among Black men, among whom the balance of risks and benefits is not definitively established but likely favor screening. Black men remain a vulnerable population and the group at highest risk of death from prostate cancer.

Decision coaching is the process by which a non-healthcare provider coach “provides a patient with individualized, nondirective guidance to meet decision-making needs in preparation for consultation” with a healthcare provider [17]. Decision coaching improves incorporation of patients’ values and goals into treatment decisions, promotes SDM, and improves communication between patients and providers [18–24]. Prior studies have employed nurses as decision coaches and demonstrated improvement in decision-making scores but are difficult to disseminate due to cost considerations [25, 26]. Other studies have attempted to employ lower-cost personnel as decision coaches such as college students [27]; however, in many instances, a decision coach with specific cultural sensitivities is required. A lower-cost, high-efficacy decision coach for prostate cancer screening is needed critically.

The community health worker (CHW) model is effective in communities requiring a culturally sensitive, contextualized approach to health promotion and intervention [28, 29]. The strength of this model is derived from the multi-theoretical roots of community organizing, social support, social networks, self-efficacy, and peer models [28, 29]. A community health worker is a public health worker that can provide liaison between health services and their community to facilitate access and optimize the quality and cultural competence of services delivered. A CHW intervention can be a low-cost approach to improve community health and well-being and can bridge the cultural and social barriers between underserved communities and the health care system [11, 28, 30, 31]. CHWs are effective in supporting cancer screening decisions in the Black community [32, 33]. In this randomized control trial, we aim to demonstrate that CHW-led decision coaching can be a feasible, low-cost intervention to improve patient decision-making and enhance provider experience.

### Objectives

#### Study aim

Our study proposes the following specific aims:

1. To test whether a CHW-led decision coaching program affects decision quality, the decision-making process, patient-provider communication, and PSA utilization for Black men in the primary care setting;
2. To assess whether a CHW-led decision coaching program improves provider experience with counseling Black men considering PSA screening;
3. To determine the cost and budget impact of a CHW-led decision coaching program for PSA screening;
4. To assess participant attitudes, behaviors, and norms around PSA screening and assess perceptions of the feasibility, acceptability, and sustainability of CHW-led decision coaching in the primary care setting.

### Study design

The study is a randomized control trial to evaluate the effectiveness of a CHW-led decision-coaching program to facilitate SDM for prostate cancer screening decisions among Black men at a primary care FQHC. We will enroll 228 Black men aged 40–69 years who are patients at the participating clinical site and the providers who care for them (*n* = 8). Patients will be randomized to either the intervention or the control arm of the study. Participants in the intervention arm will receive a decision aid by mail and will receive CHW-led decision coaching on PSA screening 1 h before their appointment, while those in the control arm will still receive the decision aid by mail but will have a CHW-led discussion on cardiovascular dietary and lifestyle modification rather than PSA screening; the discussion of cardiovascular risk reduction will serve as an attention control.

**Fig. 1:**
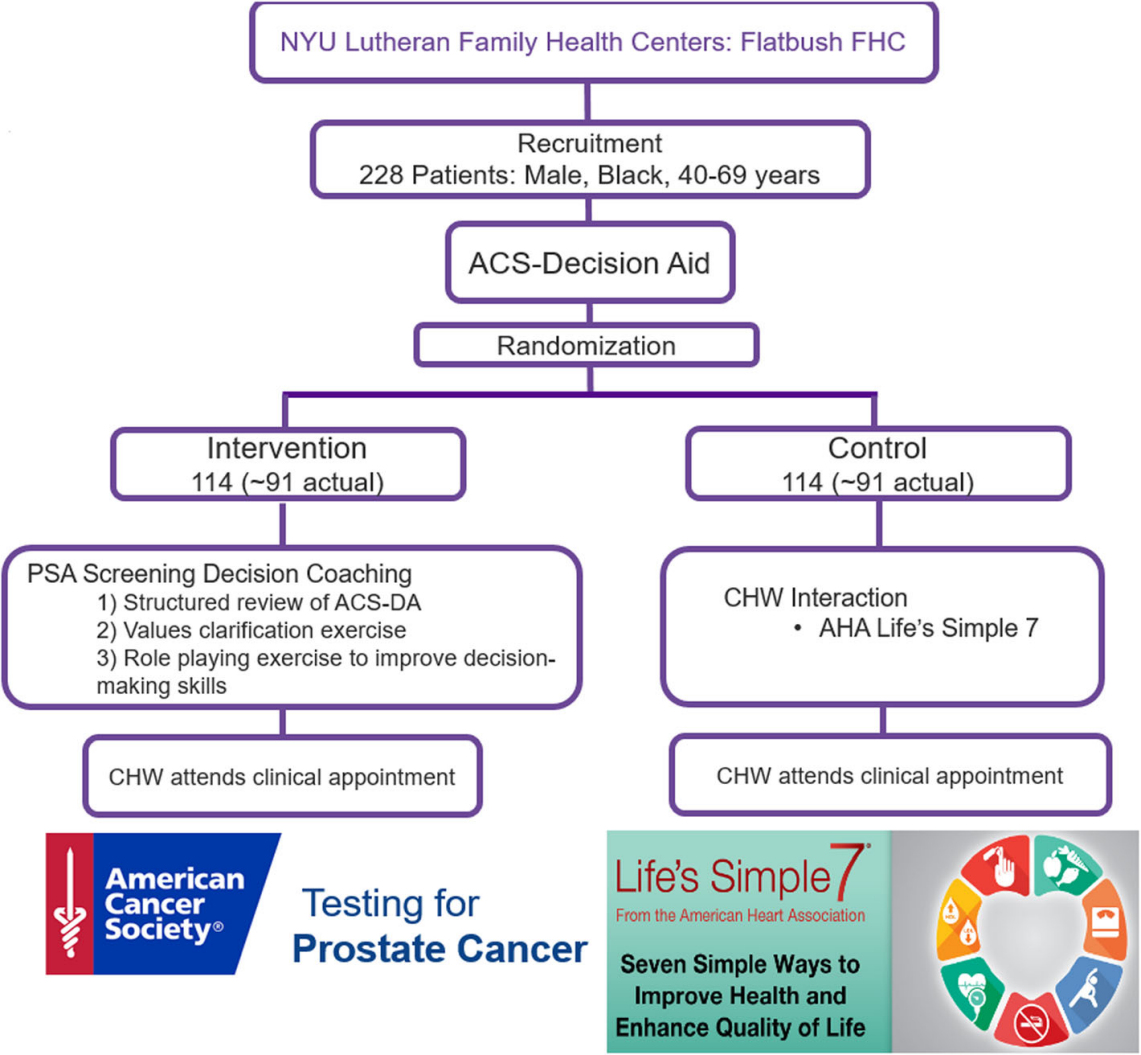
Overview of the methods

Patients will complete four surveys: (1) baseline at enrollment in the clinic prior to coaching, (2) immediately following coaching, but before provider consultation, (3) following provider consultation, and (4) at 6 months post-clinic visit. Providers at the FQHC will complete three surveys: (1) at study initiation, (2) after each patient encounter, and (3) at study completion immediately before participating in a semi-structured interview that will assess perceptions of the intervention’s acceptability and feasibility. We will explore communication in both patient and provider groups. The protocol follows the Standard Protocol Items: Recommendations for Interventional Trials (SPIRIT) guidelines and fulfills the SPIRIT checklist (see Additional file 1). An overview of the methods is shown in Fig. 1.

### Sample size calculation

In determining the recruitment target, we considered the detectable group differences in patient knowledge and decision quality based on pilot study data, differences in PSA screening rates between the control and intervention arms, and expected dropout. In our PSA pilot study, the means for knowledge change were 2.6 (SD = 2.81) and 5.1 (SD = 3.19) in the control and intervention arms, respectively. Using a two-sided *Z*-test for two proportions, a total group of 182 subjects with 91 subjects in each arm can achieve 80% power to detect a 20% difference in the post-intervention PSA screening rate between control and CHW arms at 5% type I error. Conservatively, assuming 20% dropout, 228 participants will be recruited to achieve a final sample of 182 (91/arm) patients. This sample size will allow us to be sufficiently powered to detect differences in our primary study outcomes: knowledge, decision quality, and differences in PSA screening.

## Methods

### Setting and recruitment

This study will be performed at a primary care FQHC in New York. Data collection and decision coaching will occur at this clinic. Recruitment of eligible participants will be conducted by study staff who will review weekly electronic appointment reports and call patients using an IRB-approved recruitment script.

### Participant eligibility

Consistent with prior research, we will exclude men seen within 9 months of a previous PSA test or within 180 days of a primary diagnosis of urinary obstruction, prostatitis, hematuria, disorders of prostate, unexplained weight loss, or lumbar back pain. PSA in these settings is not obtained for screening purposes [34]. Men previously diagnosed with prostate cancer (ICD-10-CM C61), and those presenting for an indication other than a well visit will be excluded.

We will enroll all eight providers who care for adult men in the FQHC: four full-time family nurse practitioners, three internal medicine physicians, and one family medicine physician. We will also enroll senior administrative staff (*n* = 2) to gain further insight on acceptability and workflow.

### Randomization, allocation, and blinding

An independent randomization process will be implemented by each provider. We will stratify the randomization by age, using two strata: 40–55 years old and 55–69 years old. Within each stratum, patients (4 person blocks) will be randomized with a computer-generated permuted block random number to the control or intervention arm upon enrollment by an independent statistician. All study staff and providers who assess outcomes will be blinded to the randomization arms. Breaking of study blinding for individual participants may occur if the research coordinator finds a critical PSA result (defined as PSA > 4) during the 6 months follow-up which has not been addressed by the provider. The study team will contact the provider immediately and urge them to have a consultation with the patient.

### Decision aid

This project will use the American Cancer Society Prostate Cancer Early Detection Decision Aid (ACS-DA) [6], which describes the prostate gland and its function, prostate-related problems (including cancer), prostate cancer risk factors and symptoms, prostate cancer early detection and screening paradigms, the pros and cons of PSA screening, and possible follow-up tests in response to abnormal PSA results. The ACS-DA has demonstrated effectiveness among medically underserved populations of Black men, among whom it substantially increased knowledge, lowered decisional conflict, and was well-accepted [35].

#### CHW intervention

In the intervention arm, the CHW coach reviews the content of the mailed decision aid and conducts a structured decision counseling session about prostate cancer screening [36]. Decision coaching will include the following components: (1) a structured discussion with the patient focusing on determining his understanding of his prostate cancer risk, screening options, and decision-making goals and values, (2) role-playing exercises to improve shared decision-making skills, and (3) coach accompaniment to the patient’s appointment.

The coach will initiate the session by asking the patient about his values and goals for screening. The coach will then ask the patient what questions the patient has and what information he needs to make his decision. The coach will ascertain knowledge and ask the patient to think through the questions he would like to ask his provider. Finally, the coach will discuss any concerns the patient may have. The coach will encourage the patient to participate in role-playing exercises, allowing the patient to practice talking with his provider about treatment values, goals, and preferences.

The patient will be encouraged to make a final decision about PSA screening in consultation with his provider. At the conclusion of the coaching session, the patient and provider will receive a document summarizing the content of the interview which prioritizes questions, values, and goals. The visit will be audio-recorded to assess coaching fidelity and the quality and content of the discussion.

#### Control arm

Participants in the control arm will receive, but not review with a CHW, the content of the mailed decision aid. CHW will encourage discussion of the decision aid with their provider if they inquire. The coach will offer general health coaching using an educational tool focused on dietary and lifestyle modification to reduce the risk of cardiovascular disease. Specifically, the coach will use the American Heart Association’s “Life’s Simple 7” educational tool [37]. Health coaching will focus on four modifiable health behaviors including smoking cessation, healthy diet, physical activity, and body mass index (BMI), and three modifiable biological factors, including blood pressure (BP), total cholesterol, and fasting glucose, as described in the control educational tool. This arm consists of the patient choosing preferred topics out of the 7 options provided. This component will serve as an attention control for the personal interaction experienced by men in the intervention arm. The coach will accompany control patients to their appointments and take notes. The visits (both intervention and control) will be audio-recorded to assess the quality, fidelity, and content of the discussion.

### Outcome assessment

Outcome measurement will be based on surveys, interviews, and data collected from the medical records by trained community health workers. Primary, secondary, and exploratory outcomes are summarized in Table 1.

**Table 1:** Primary, secondary, and exploratory outcomes

Study endpoints/outcomes	
Primary study outcomes	
The following are primary study outcomes:	
● Decision quality: Measure of informed choice to evaluate screening decision and attitudes towards the screening test	
● Patient knowledge: Patient knowledge of prostate cancer and PSA screening	
● PSA screening rates: PSA rates collected through patient self-reported PSA testing and EHR data on PSA test utilization	
Secondary Study Outcomes	
Secondary study outcomes are:	
● Perception of quality of care: Patient perception of quality of care assessed through domains of communication, decisional self-efficacy, self-efficacy in communicating with their provider, satisfaction and decisional conflict	
● Experience with decision coaching program: Provider experience with decision coaching program measured through encounter satisfaction, difficulty, and communication	
Exploratory outcomes	
● Net cost of CHW-led decision coaching program for PSA screening: cost of CHW-led decision coaching program and its effect on prostate cancer screening costs by measuring CHW program costs and healthcare utilization costs	
● Behaviors and norms around PSA screening and perceptions of feasibility and acceptability of CHW-led decision coaching: Qualitative evaluation of patient and provider perceptions through in-depth, semi-structured interviews and other qualitative data; triangulation of mixed methods data sets from patients and provider interviews, clinical encounters, and surveys to better understand implementation.	

### Primary outcomes

#### Decision quality

We will objectively measure decision quality using two domains as defined by Sepucha et al.: (1) being informed (e.g., accurate understanding about screening and its risks and benefits) and (2) making preference-concordant decisions (i.e., treatment consistent with patient preferences as determined by responses to survey questions) [38]. Measurement of these domains will be operationalized using the following tools and techniques:

- The Measure of Informed Choice includes 4 items assessing attitudes towards the screening test and a record of test uptake [39].
- The Decisional Balance Scale will assess the patient’s attitudes towards PSA testing. The scale consists of a six-item pros scale (alpha = 0.87) and six-item cons scale (alpha = 0.82) scored on a 5-point Likert scale (strongly agree to strongly disagree) [40].
- Knowledge will be measured using a survey developed by our group that assesses understanding of prostate cancer and PSA screening [41]. The survey was piloted among Black men recruited from churches in Harlem, New York, enrolled in a study to help determine whether PSA screening was right for them.
- PSA screening rates will be collected 6 months post-intervention through self-reported PSA testing and extracted EHR data on PSA test utilization. The association between PSA utilization and intervention exposure will then be tested.

### Secondary outcomes

Patient perception of quality of care will be assessed through the domains of communication, decisional self-efficacy, self-efficacy in communication with their provider, satisfaction, and decisional conflict.

- *Communication*:
  - The Combined Outcome Measure for Risk communication and treatment Decision-making Effectiveness (COMRADE) is a 20-item measure validated for clinical encounters [42]. Sub-scales include (1) satisfaction with physician communication and (2) patient confidence in the decision. The scale has excellent internal consistency (Cronbach’s *α* = 0.92).
  - The Questionnaire Concerning the Doctor-Patient Communication Skills [43] is a validated 19-item scale capturing the process (greeting, listening) and content (explanations and next steps) aspects of the visit from the provider’s and patient’s perspectives.
- The Decision Self-Efficacy Scale is an 11-item scale measuring self-efficacy to perform informed decision-making (e.g., getting needed information, asking questions, expressing opinions, and asking for advice) [44].
- The Perceived Efficacy in Patient-Physician Interactions is a 10-item scale measuring self-efficacy for provider communication [45]. The scale is reliable (Cronbach’s *α* = 0.91) and valid in older adults.
- The Satisfaction with Decision Scale is a 6-item measure assessing patient satisfaction with their decision and decision-making process [46].
- The Decisional Regret Scale is a validated, 5-item scale measuring regret or remorse following a health care decision [47].
- The Decisional Conflict Scale will measure patients’ perceptions of uncertainty in choosing options, feelings of having adequate knowledge and clear values, and effective decision-making [48].
- Patient experience with the decision-coaching program will be assessed using a survey developed by our group that measures the usefulness as well as the impact of the CHW intervention on the patient and the appointment.
- Provider experience with the decision-coaching program will be measured through encounter satisfaction, difficulty, and communication.
- The Physician Satisfaction Scale designed for encounter-specific situations measures satisfaction [49]. The survey has 2 dimensions and 16 items measuring understanding of the patient’s problem, perceiving patient comprehension, and affective reactions. Internal consistency is good (Cronbach *α* = 0.85).
- The Mental Work-Load Instrument assesses difficulty with the subjective experience or cost incurred by a physician in performing patient care [50]. The survey has 5 dimensions and 6 items addressing mental effort, physical effort, difficulty, performance, and stress. Internal consistency is good (Cronbach *α* = 0.80).
- Provider communication will be assessed using the Questionnaire Concerning the Doctor-Patient Communication Skills [43].

### Exploratory outcomes

Exploratory outcomes include the comparative cost of the intervention and behaviors and norms surrounding PSA screening. The cost of a CHW-led decision-coaching program and its effect on prostate cancer screening costs will be measured by analysis of the CHW program costs and healthcare utilization costs. Intervention costs will be evaluated using the methods described by Ritzwoller et al. [51], which documents and assesses the components of the intervention and its implementation, specifies a short-term time horizon relevant to potential decision-makers who might adopt the intervention if proven effective, and outlines important sensitivity analyses for the cost analysis. We will capture CHW time associated with the intervention and apply relevant labor rates (wage plus fringe) from the Bureau of Labor Statistics. We will summarize healthcare utilization costs that emanate directly from the intervention. These include estimating costs for select screening procedures from pre-specified diagnosis and procedure codes [G0103, 84152, 84153, and 84154]. Screening-related activities will be ascertained beginning with the initial clinic visit and any related care as follows: costs for PSA screening; prostate biopsies performed within 180 days after a PSA test; and hospitalizations due to biopsy complications, defined as those that occurred within 30 days of prostate biopsies and have ICD-10 primary diagnosis codes consistent with complications. We will apply New York State Medicaid reimbursement rates as a lower bound estimate of healthcare costs and national average Medicare reimbursement rates as an upper bound estimate of healthcare costs.

Behaviors and norms surrounding PSA screening will be measured using qualitative methods to identify and describe the attitudes and perceptions of Black men and their providers regarding PSA testing, the CHW-led decision-coaching intervention, and SDM. In-depth, semi-structured interviews with both patients and providers will be conducted. The interviews will be audio-recorded and transcribed for thematic analysis.

#### Data collection

To evaluate the efficacy of CHW-led decision coaching, we will collect quantitative, qualitative, and cost data.

Quantitative data will be collected from (1) surveys administered to patients and providers as well as from (2) patient clinical data collected from the institution’s electronic health record, Epic Electronic Medical Record (Epic Systems Corporation).

**Fig. 2:**
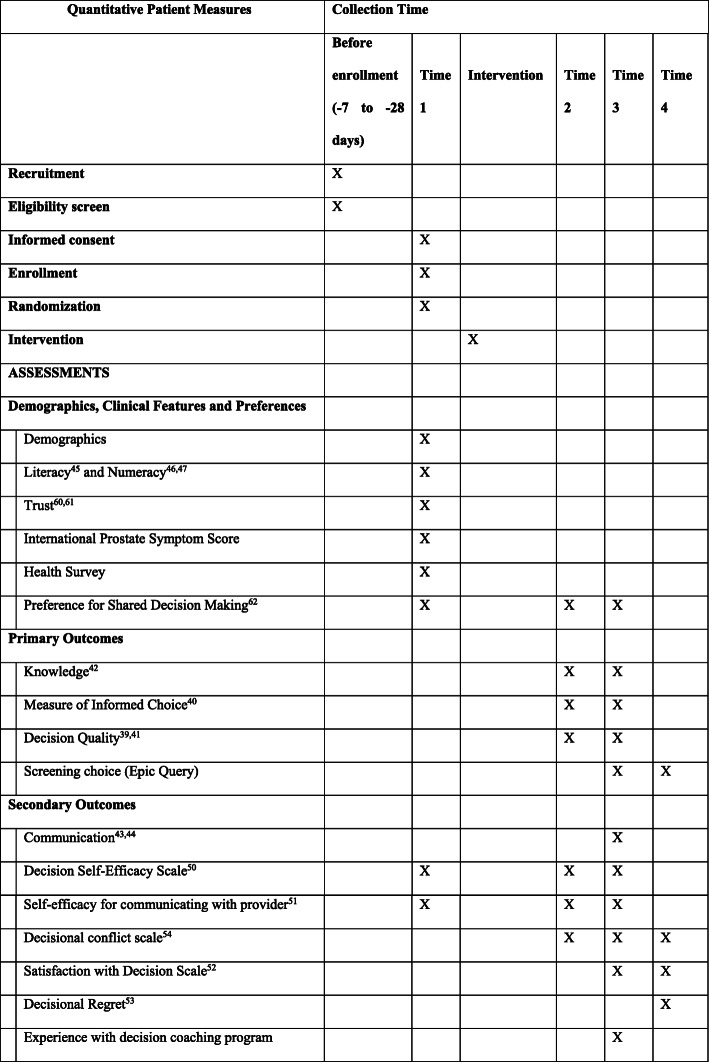
Quantitative patient measures

Figure 2 shows the SPIRIT schedule of assessments and interventions for study patients. Time 1 measures are completed at enrollment in the clinic before coaching. Time 2 occurs after coaching but before provider consultation. Patients will complete Time 3 measures following their consultation. Time 4 measures are collected 6 months post-clinic visit. A subset of patients will be recruited to complete a qualitative interview at Time 4.

**Fig. 3:**
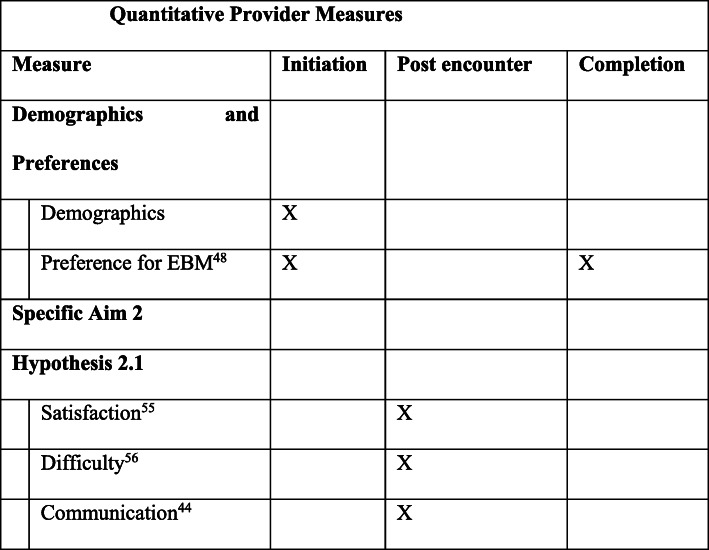
Quantitative provider measures

The SPIRIT schedule of assessments for providers is shown in Fig. 3. Providers at the FQHC will complete measures at three time points. Basic demographic information will be collected at study initiation. Measures will be collected via brief survey after each patient encounter (the provider will be blinded as to whether the patient was in the control or intervention arm). A final survey will be completed at study completion immediately before the semi-structured qualitative interview.

To promote participant retention in follow-up procedures, we will reimburse both patient and providers for their participation in the program and completing follow-up assessments.

#### Data management

The study case report form (CRF) is the primary data collection instrument for the study. All data will be entered into a secure, HIPAA-compliant, digital data collection system, REDCap. All data fields will be entered using free text and multiple-choice entries with reminders to ensure fields are entered appropriately. REDCap has data auditing to ensure any changes to data are recorded and justified. Hard copies will be kept in a locked storage area in a locked office and building. Access to study records will be limited to IRB-approved members of the study team.

A Data Safety and Monitoring Board (DSMB) will be established to ensure the safety of participants and the validity and integrity of the data. The DSMB will consist of a statistician (distinct from project statistician) and two pedagogical experts (distinct from study personnel). The DSMB will approve the Data and Safety Monitoring Plan and meet annually to assess overall compliance with the IRB-approved protocol. Other responsibilities of the DSMB include evaluating the progress of the study including assessments of data quality, participant risk versus benefit, and other factors that affect study outcome; making recommendations to study investigators and IRB to ensure protection of human subjects concerning continuation or conclusion of the trial; and protecting study data confidentiality.

The principal investigators are responsible for reporting adverse events to the NYU IRB, the entity granting IRB approval. Adverse events (AEs) and serious adverse events (SAEs) that are severe in nature and could potentially affect the well-being of study participants will be reported to IRB immediately. Also, the contact-PI (Dr. Makarov) will summarize AEs and SAEs in annual continuation reports.

### Statistical analysis

An “intent-to-treat” (ITT) approach will be used. We will examine (1) comparability of study arms at baseline (based on chi-squared statistics or *t*-tests, as appropriate), (2) relationships between the response variables and potential covariates, and (3) predictors of missing data/dropout.

We will use linear mixed models for continuous outcomes, logistic generalized linear mixed models for binary outcomes, and random effects multinomial models for outcomes with more than 2 levels, such as adherence. In all models, time (2 dummy variables) and intervention will be included as fixed effects; provider will be a random effect. The intervention effect of interest is the treatment X time interaction. Identified predictors of missing data will be included as covariates in this random effect’s framework, to provide unbiased estimates of the intervention effect under an assumption of missing at random (i.e., missingness depends only on observed—not on unobserved—covariates). We will conduct sensitivity analyses to assess plausible departures from this assumption. Other demographic and clinical covariates will be included as necessary in adjusted analyses. Model assessment will be conducted using appropriate regression diagnostics. The primary and secondary analyses will be done using Stata and SAS, and MPlus will be used in the mediation analyses.

Specifically, to determine the effect of the intervention on the primary outcomes, a random effects (generalized) linear regression model will be used to test absolute and time-specific differences attributable to the intervention. In additional analyses, we will adjust for other covariates which may be unbalanced between the intervention arms at baseline at *p* = 0.10. For hypotheses pertaining to patients’ perception of care quality, we will model satisfaction with provider communication, self-efficacy, difficulty, and decisional conflict in separate analyses of control and intervention arms to examine possible associations between those measurements and the outcomes.

We will summarize costs using descriptive statistics, including mean, medians, and standard deviations, for each intervention component including downstream costs outlined above. We will tally all costs to estimate the intervention’s budget impact and the total average cost per patient. We will conduct extensive sensitivity analyses, including a range of relevant salaries for providers and staff and upper and lower bounds for estimating the cost of healthcare utilization.

### Limitations

It may not be possible to blind providers completely, especially with the CHW Decision Coach’s presence at the clinic visit. The discussion between coach and patient may reveal their previous interaction. Providers may thus be prompted to engage in greater SDM than they might have outside of the study. Providers will also be handed a document with a prompt to identify the patient as part of the study and to remind the provider to engage in a discussion of prostate cancer screening during the visit. This is likely to spur SDM type discussions in both control and interventions arms, biasing our results towards the null and making a type I error less likely.

## Discussion

In this trial, we evaluate the efficacy of a CHW-led decision-coaching program to facilitate shared decision-making for PSA screening among Black men at a primary care FQHC. CHW-led interventions are known to improve awareness, knowledge, support, and efficacy to reduce the impact of chronic disease and cancer in underserved populations [52–56]. Thus, CHWs seem ideally suited as decision coaches in primary care practices seeking to facilitate SDM for PSA screening among Black men, which has not been tested previously. A rigorous study design will be applied to this question with an independent randomization process and blinding of all individuals who assess study outcomes, including healthcare providers, the statistician, to reduce bias.

### Trial status

This trial is ongoing. Recruitment began on June 19, 2019, and will continue until September 2021. The trial procedures are expected to be completed by the end of March 2023.

Protocol version 02.26.20.

## Supplementary Information

**Additional file 1.**

## Data Availability

This study will comply with the NIH Public Access Policy, which ensures that the public has access to the published results of NIH funded research. It requires scientists to submit final peer-reviewed journal manuscripts that arise from NIH funds to the digital archive PubMed Central upon acceptance for publication.

## Abbreviations

CFR: Code of Federal Regulations
CHW: Community health worker
CRF: Case report form
DSMB: Data and Safety Monitoring Board
FQHC: Federally Qualified Health Center
HIPAA: Health Insurance Portability and Accountability Act
IRB: Institutional Review Board
*N*: Number (typically refers to participants)
NIH: National Institutes of Health
PSA: Prostate-specific antigen
SDM: Shared decision-making
US: United States
